# Schlesinger Nailed It! Assessing a Key Primary Pharmacodynamic Property of Phages for Phage Therapy: Virion Encounter Rates with Motionless Bacterial Targets

**DOI:** 10.3390/ddc2030034

**Published:** 2023-08-18

**Authors:** Stephen T. Abedon

**Affiliations:** Department of Microbiology, The Ohio State University, Mansfield, OH 44906, USA

**Keywords:** bacterial aggregates, bacterial clusters, bacteriophage therapy, biofilm, phage therapy, phage-antibiotic synergy

## Abstract

Bacteriophages (phages) are viruses of bacteria and have been used as antibacterial agents now for over one-hundred years. The primary pharmacodynamics of therapeutic phages can be summed up as follows: phages at a certain concentration can reach bacteria at a certain rate, attach to bacteria that display appropriate receptors on their surfaces, infect, and (ideally) kill those now-adsorbed bacteria. Here, I consider the rate at which phages reach bacteria, during what can be dubbed as an ‘extracellular search’. This search is driven by diffusion and can be described by what is known as the phage adsorption rate constant. That constant in turn is thought to be derivable from knowledge of bacterial size, virion diffusion rates, and the likelihood of phage adsorption given this diffusion-driven encounter with a bacterium. Here, I consider only the role of bacterial size in encounter rates. In 1932, Schlesinger hypothesized that bacterial size can be described as a function of cell radius (R, or $R^{1}$), as based on the non-phage-based theorizing of Smoluchowski (1917). The surface area of a cell—what is actually encountered—varies however instead as a function $R^{2}$. Here, I both provide and review evidence indicating that Schlesinger’s assertion seems to have been correct.

## 1. Introduction

Phage therapy is the use of bacterial viruses to treat infections caused by bacteria [1–3]. We can consider this phage therapy from the perspective of pharmacology [4], with pharmacology traditionally differentiated into what is labeled as pharmacokinetics vs. pharmacodynamics. Pharmacokinetics [5,6] describes what influences the ability of a drug, in this case an antibacterial agent, to reach sufficient concentrations in situ to have a pharmacologically useful effect. Typically, this is conceptualized in terms of the impact that the body has on the drug, either causing in situ drug concentrations to increase—such as those associated with drug absorption into the blood and then distribution throughout the body—or to decline. The latter can be due to drug inactivation (a consequence of what is termed in pharmacokinetics as metabolism) or instead excretion from the body, the latter such as via the action of the kidneys. Resulting drug concentrations are relevant especially as they are achieved in the vicinity of targeted tissues.

Pharmacodynamics [6,7], contrasting pharmacokinetics, describes the impact of a drug on the body. In addition, though, this can be in terms of the impacts of antibacterial agents on bacteria, especially as may be achieved by a given drug concentration found in the vicinity of those bacteria. For phages as antibacterial agents, particularly given phage-particle one-hit killing kinetics [8], pharmacodynamic processes can be distinguished into phages first adsorbing and then their killing resultingly phage-infected bacteria. The rate that bacteria become adsorbed is, in turn, a function of a combination of phage concentrations and what is known as the phage adsorption rate constant [9–12].

I focus here on this latter component of phage therapy pharmacology. The emphasis is on how phage encounters and therefore adsorption rates may vary with bacterial cell size, specifically with two-fold increases in bacterial diameter likely resulting in only two-fold increases in phage adsorption rates, that is, rather than, e.g., four-fold increases. That phage adsorption rates should vary with the radius of a bacterium (R), rather than with radius squared $\left(R^{2}\right)$, is consistent with the theorizing of Schlesinger [13,14], as published in 1932, but which here I corroborate based upon a combination of in silico modeling (which I provide also as an online-accessible app), reexamination of experimental data provided by Hadas et al. [15], review of additional (and more sophisticated) modeling provided by Eriksen et al. [16], and also dimensional analysis. This ‘rule’ that phage encounter rates with bacteria should vary as a function essentially of bacterial width rather than bacterial surface area likely can be extended to consideration of phage encounter rates with spherical bacterial clusters [16], e.g., such as can be found in association with bacterial infections of cystic fibrosis lungs [17].

## 2. Some Background

The magnitude of phage adsorption rate constants (k) can be derived from theory as

(1) k=4πRCf,

where R is the radius of a phage-targeted bacterium, C refers to virion diffusion rates (often abbreviated instead as D), and f is the likelihood of virion attachment to a bacterium given encounter. Thus, k is considered to be a function of the radius of involved bacteria, R [9,10,13,14], and this is rather than a direct function of its surface area.

This radius represents the size of just the targeted bacterium, and not also that of the phage. The reason for this emphasis on bacterial rather than virion size is that bacteria typically are large relative to phage virions [10]. For example, envision an arrow encountering a target, where it is the target’s size rather than that of the much smaller arrow that generally is crucial in determining whether the arrow will ‘find its mark’. Hitting a target—here, the bacterium—intuitively thus should be a function of the area of a target rather than just its radius. Area in this case could be either that of a circle as a cross section of a sphere $\left(\pi R^{2}\right)$ or instead the surface of that sphere $\left(4\pi R^{2}\right)$, thus being functions of $R^{2}$ rather than of just $R\;\left(=R^{1}\right)$. In addition, note that the calculation for k as provided in Equation (1) also includes the terms 4π [18], further confounding the issue of why $R^{1}$ rather than $R^{2}$ is being used in that equation.

Why then has $R^{1}$ been used to describe the size of bacteria in adsorption rate constant calculations rather than something containing instead the term, $R^{2}$? To address this issue, one needs to go back to the 1917 article of Smoluchowski [18] on an “Attempt at a mathematical theory of the coagulation kinetics of colloidal solutions”. By “coagulation”, what is meant is the joining together of two previously not joined particles (“From this moment on, the pair in question should form an indivisible whole as a result of aggregation”, p. 137). These particles are otherwise diffusing, but when they collide, they are converted from two particles, each consisting of “a sphere of attraction of radius R” (p. 137, all translations are by Google), into a single particle. The following sentence (from the same page) then states, “From now on it will be his Brownian molecular movements undisturbed in a normal way only up to the point where—precisely as a result of those motions—the center of another particle enters its sphere of attraction.” Notably, Schlesinger [13,14] then applied these ideas of Smoluchowski to consider the interactions of phages with bacteria. See too Delbrück [19] who summarizes Schlesinger’s theory along with the more recent analyses presented by Berg and Purcell [20].

I began the analysis presented here based on a hunch that the use of $R^{1}$ to predict a phage’s adsorption rate constant could represent a bit of an accident of history, one of assuming equivalence of potentially non-analogous systems and this is rather than $R^{1}$ with certainty representing a rigorously meaningful measure of phage-bacterial interactions. Furthermore, chemical collision theory defines collision frequencies (Z), such as between gas molecules, as proportional (∝) instead to $R^{2}$, described as a collisional cross section. The associated collision frequency equation also supports the idea that virion size may be ignored relative to that of the much larger bacteria in determining collision rates [21], i.e.,

(2) $$Z\propto PN{\left(R_{P}+R_{N}\right)}^{2},$$

where P and N are the concentrations of two different entities while $R_{P}$ and $R_{N}$ represent the radii of the two corresponding particles of differing sizes, such as perhaps the radius of a phage virion $\left(R_{P}\right)$ along with that of the target bacterium $\left(R_{N}\right)$. Consequently, this review began as an effort toward testing the validity of Schlesinger’s assertion that frequencies of collisions with bacteria should be a function of $R^{1}$ rather than of $R^{2}$.

Notwithstanding my early hypothesizing that Schlesinger [13,14] could have been wrong, one employs $R^{1}$ rather than $R^{2}$ when considering particle collision rates generally, that is, beyond the realm of just phages and bacteria. The general case, though, typically involves both particles moving [22] and this is rather than just one particle moving but not the other, i.e., rather than a moving phage virion encountering what are assumed to be stationary, that is, motionless bacteria [10] (for review instead of the impact of bacterial motion on phage adsorption kinetics, see [12]). Schlesinger [13,14] for instance described diffusion in his model as only involving phages. This use of just $R^{1}$ can be seen particularly in the derivation of what is known as a collision kernel [22], of which phage adsorption rate constants can be viewed as a special case. Another deviation from basic particle collision theory is that bacteria can be much larger than phages, such as ten or more times larger.

The basis of the current investigation thus was that of my questioning, perhaps naïvely, the validity of $R^{1}$ serving to derive k rather than, for example, $R^{2}$, as relevant to our theoretical understanding of phage therapy pharmacodynamics. To do this, I generated a JavaScript-based and online-available app in which a phage random walk is simulated. This model makes no assumptions as to how representation of the size of a bacterial cell, e.g., $R^{1}$ vs. $R^{2}$, may affect the number of program steps a randomly diffusing virtual phage may take before encountering a simulated bacterium (see encounter.phage.org for the version of this program used to run the simulations described here). Ultimately, as I show, this model provides a corroboration of $R^{1}$ serving as the basis of phage adsorption rates, at least with idealized spherical bacteria. I then review additional, published evidence suggesting that Schlesinger’s [13,14] use of $R^{1}$ rather than $R^{2}$ in mathematically deriving phage adsorption rate constants not only appears to have been quite reasonable but likely can be extended to beyond predicting rates of phage encounter with simply spherical, individual bacteria.

## 3. In Silico Exploration

Causality refers to the dependence of some output on a given input. That implies some degree of determinism, i.e., such that outputs are constrained in some manner by inputs. Stochasticity, i.e., randomness, need not be absent given causality, however, and what is known as a Markov process explicitly combines determinism and stochasticity, i.e., where outputs are constrained but still not completely predetermined. A random walk is a Markov process in that location at time, t, is dependent on location at time, t−1, whatever units of time may be used. Particularly if intervals are short enough and velocity is sufficiently slow, then the specific location of, e.g., a diffusing object at time, t+1, will be a function in part of its specific location at time, t.

A phage undergoing diffusion is subject to a random exploration of its fluid environment. The velocity of that exploration is a function of a virion’s diffusion rate, with Equation (1) telling us that the faster that a virion diffuses then the more likely that encounter will occur with a bacterium over a given span of time. In nature, such diffusion is not a discrete process, i.e., with the direction as well as the speed with which movement occurs over even small time intervals varying across a continuum. If we constrain those movements, however, in terms of direction as well as speed, then this still is a Markov process, just with fewer possible outcomes associated with each interval of motion.

Movements, for example, can be constrained to no more than six possible directions, corresponding to plus vs. minus over the x, y, or z axes, with direction of movement at time, t−1, not impacting direction of movement at time, t. The step size of movement is also constrained, to one unit, i.e., +1 or −1. These assumptions allow for a relatively easily programmed discrete model. Thus, to explore how phage rates of encounter may vary with bacterial size, I have simulated the movement of a one-unit-size phage randomly moving one unit per time step within a three-dimensional cube (see the noted encounter.phage.org). The goal of this effort was to determine how rates of phage encounters with an immobilized bacterium may vary with cell size in the course of such a random walk—here using an idealized, spherical bacterial cell. No assumptions thus were made concerning *how* such rates of encounter may vary with cell size. As this is a stochastic process, i.e., with different outcomes for different runs despite identical starting conditions, a Monte Carlo method was employed. This is described here as averaging multiple technical repeats of in silico ‘experiments’.

### 3.1. Simulation Approach

A motionless spherical target of radius, R, as representing the bacterium, is placed in the center of a virtual cube of finite dimensions, e.g., such as 2500 phage-sized units on edge. A virtual phage is then seeded at one of this cube’s corners from which it begins its random walk. This random walk occurs as indicated above but with movement at the cube’s boundaries limited to steps that would retain the phage inside of the cube.

The distance (D) between the phage position and the center of the cube during this random walk is repeatedly calculated using standard methods,

(3) $$D=\sqrt{{\left(x_{P}-x_{C}\right)}^{2}+{\left(y_{P}-y_{C}\right)}^{2}+{\left(z_{P}-z_{C}\right)}^{2}},$$

where the subscript, ‘P’, refers to the position of the phage while the subscript, ‘C’, refers to the center of the bacterial cell. When, over the course of a phage’s random walk, D comes to be less than R(D<R), then encounter of the phage with the bacterium is assumed to have occurred [16,23].

That approximation, i.e., D<R, is slightly crude given that this is occurring within a discrete grid whereas R is not defined by the grid. That issue should be particularly problematic when cell sizes are small relative to grid sizes. Therefore, the results presented (Sections 3.1 and 3.2) are relative to a minimum cell size of radius, 10, rather than, e.g., a radius of 1, where the latter as noted would be equal to the size of an individual phage particle. Rates of movement are 1 unit per step, i.e., such that a 1 × 1 × 1 dimensioned phage moving solely linearly would span 10 units in a minimum of 10 virtual steps.

The virtual phage thus moves no more and no less than 1 unit per step, with movement constrained within the virtual cube (the “diffusion environment”), and continues the resulting random walk until its distance from the center of the virtual bacterium is less than the bacterium’s radius (R). At this point, the individual simulation ends and the number of prior steps required is recorded. The actual simulation output is the number of steps averaged over a specified number of technical repeats, such as 100 or 200, which means that the simulation is repeated that number of times for each cell size considered.

### 3.2. Comparing $R^{1}$ and $R^{2}$

**Figure 1.**
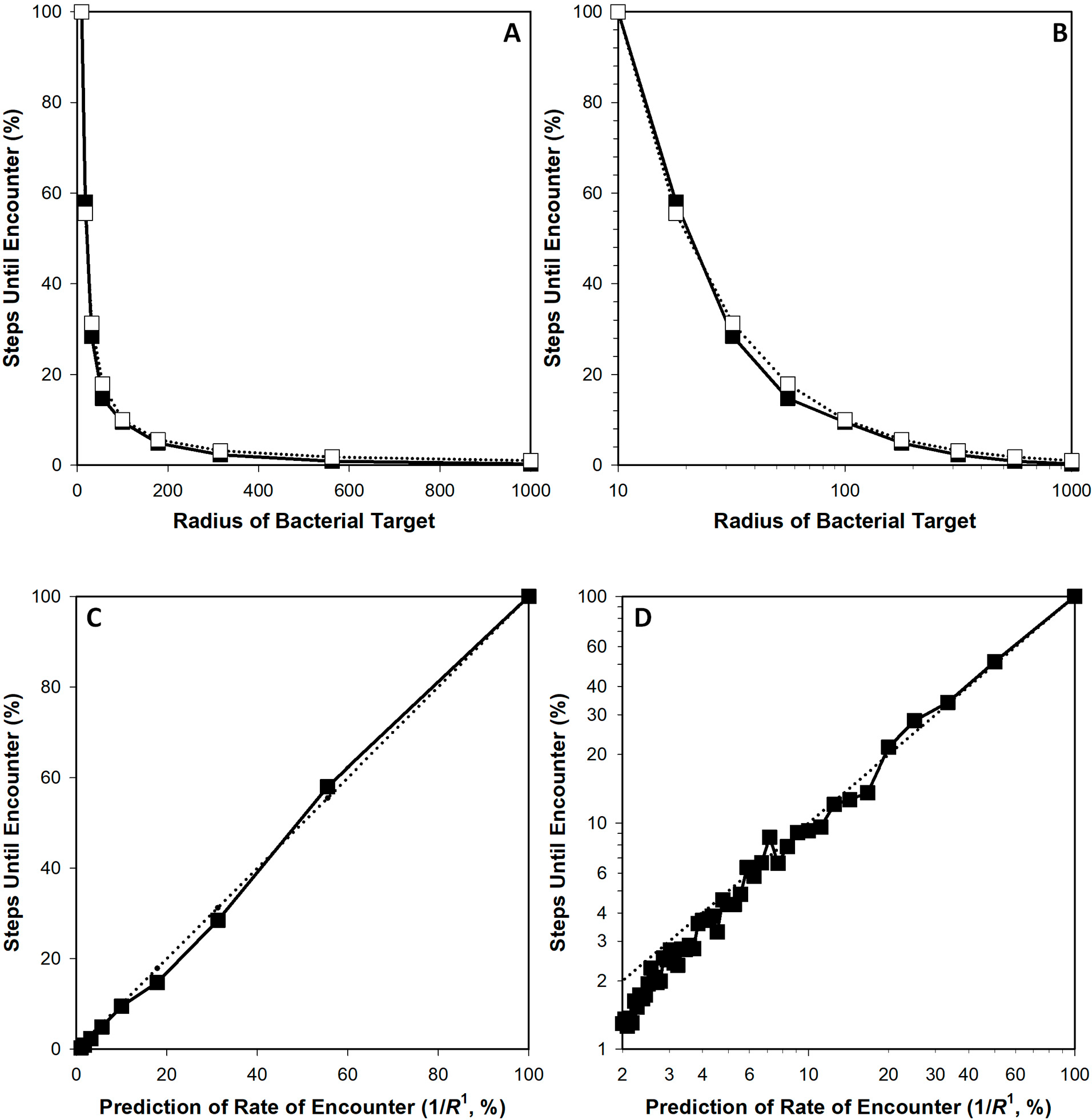
Comparison of simulation results to predictions, the latter as based on k varying with $R^{1}$. Upper panels (**A**,**B**): the same simulation shown with different x-axis scaling. The solid line with solid circles is the simulation (presented as a percentage relative to R=10 averaged results) whereas the dotted line with open squares is the prediction ($=1/R^{1}$, also presented as relative to R=10; see the main text for justification of the use of both). For this simulation, the size of the cube-shaped diffusion environment was set to 2500 units in width (vs. up to 1000 units for R of the target) and per datum there were 100 technical repeats. Lower panels: panel (**C**) is equivalent to what is shown in panels (**A**) and (**B**) except with $1/R^{1}$ divided by 1/(10 units), presented as a percentage, defining the x axis, and with the $1/R^{1}$-derived dotted-line curve shown without symbols for the sake of clarity. Here, the slope is calculated as 1.0184 with a correlation coefficient of 0.9990. Panel (**D**) is a different simulation run with cell sizes incremented up by units of 10, to R=500 units, rather than being incremented exponentially. In this case, the slope is 1.0181 while the correlation coefficient is 0.9986.

In running these simulations, the average time that is required for a phage to reach a bacterium, what can be described as a phage mean free time, can be defined as equal to 1/(kN) [24], where N is the concentration of targeted bacteria. From Equation (1), this duration thus would be predicted to be proportional to $1/R^{1}$ (for k∝R), while otherwise holding N,C, and f constant in terms of phage adsorption rates. Comparisons of the number of steps of phage movement prior to bacterial encounter, as determined from representative simulations, are provided in Figure 1 as relative to this $1/R\left(=1/R^{1}\right)$ predicted value. Note the near-identity seen between prediction (dotted-line curves) and simulation output (solid lines).

**Figure 2.**
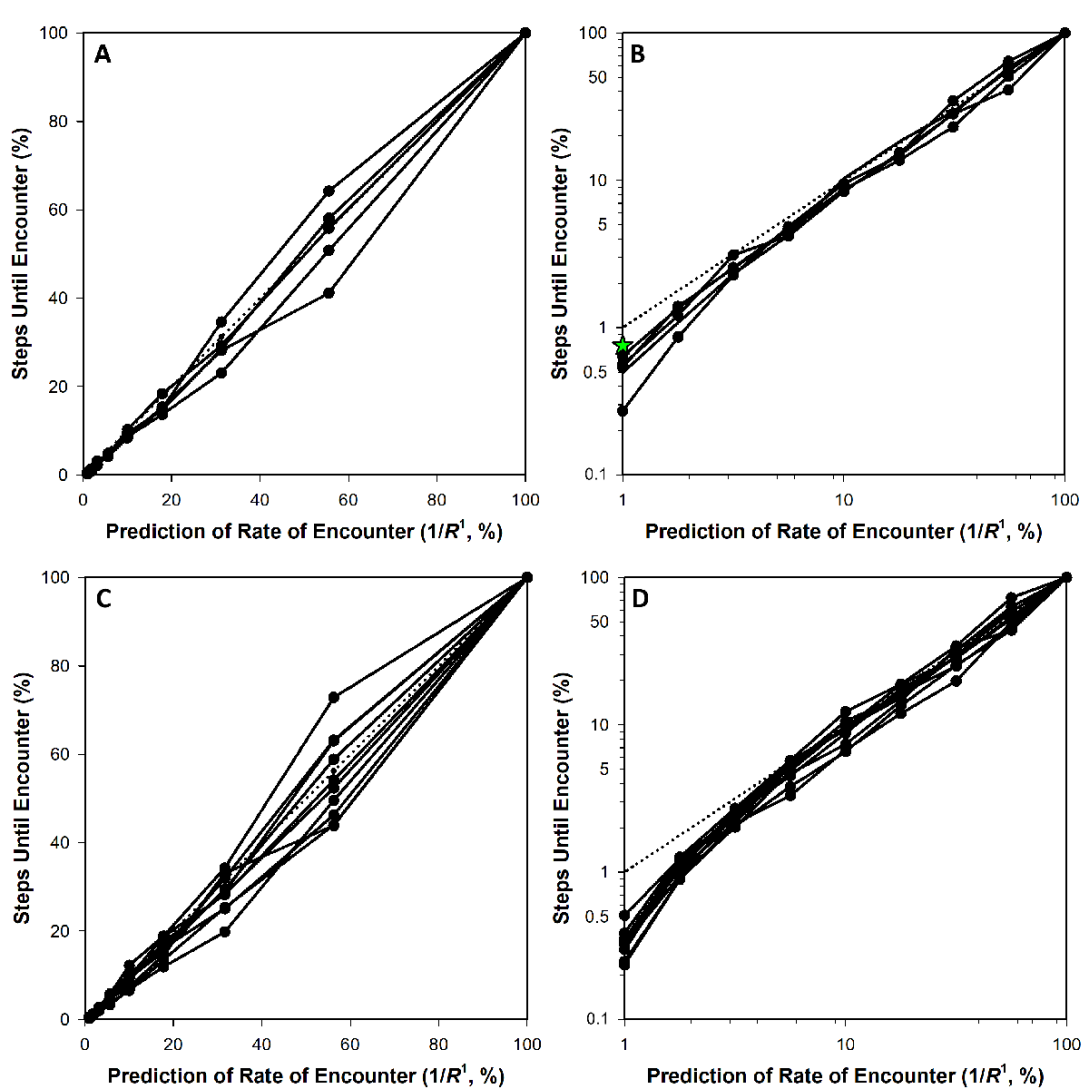
Impact of longer simulation times. Panels (**A**) and (**B**) are identical except for their axes and are based on simulations equivalent to those of Figure 1 (panels (**A**–**C**)), except for use of a diffusion environment that is 5000 units on a side (vs. 2500 units). Five separate runs are shown, keeping in mind that all data points nonetheless are independent of one another. In addition, in panel (**B**) a green star has been placed representing an averaging of ten runs within a diffusion environment of 10,000 units in size (average number of steps for five runs for a cell size of radius 1000 divided by average number of steps for five runs with a cell size of radius 10). Panel (**C**) is identical to (**A**) (diffusion environment 2500 units on a side) except graphed are ten different simulations (see also Table 1), whereas panel (**D**) is the same as panel (**C**) except with modified axes. For panel (**A**), the averaged regression slope is 0.9919 with a y intercept of −1.2867, while the correlation coefficient is 0.9950 (the dotted line is not the regression slope but instead the $R^{1}$-based prediction). As a function of $R^{2}$ (not shown, and rather than of $R^{1}$), the equivalent numbers are 0.9732 (slope), 7.9400 (y intercept), and, for the correlation coefficient, 0.9682. Though not presented, starting phages at random positions within the diffusion environment rather than at a corner does not appear to reduce the illustrated deviation from prediction of phage encounter rates with lager cells.

Note alternatively that at the largest cell sizes tested in fact rates of encounter appear to trend toward slightly faster than as predicted by $1/R^{1}$ (lower left of Figure 1D as well Figure 2D, the latter as based on simulations also considered in Table 1), with the number of simulation steps thus dropping below the dotted-line prediction. An obvious possibility would be that this deviation is a consequence of excessive crowding of the, e.g., 2000-unit diameter cell (R=1000) within a 2500-unit dimensioned, cube-shaped adsorption environment. Increasing the size of that environment to 5000- (Figure 2B) or even ten-thousand units on a side (Figure 2B, star), however, did not result in complete reversal of that trend. Therefore, tentatively, it appears that deviation from the $1/R^{1}$ prediction might occur with larger target sizes, though this was not pursued further due to computational limitations, i.e., each 5000-unit simulation (each curve presented in Figure 2A,B) required approximately 24 h of computer time to run.

**Figure 3.**
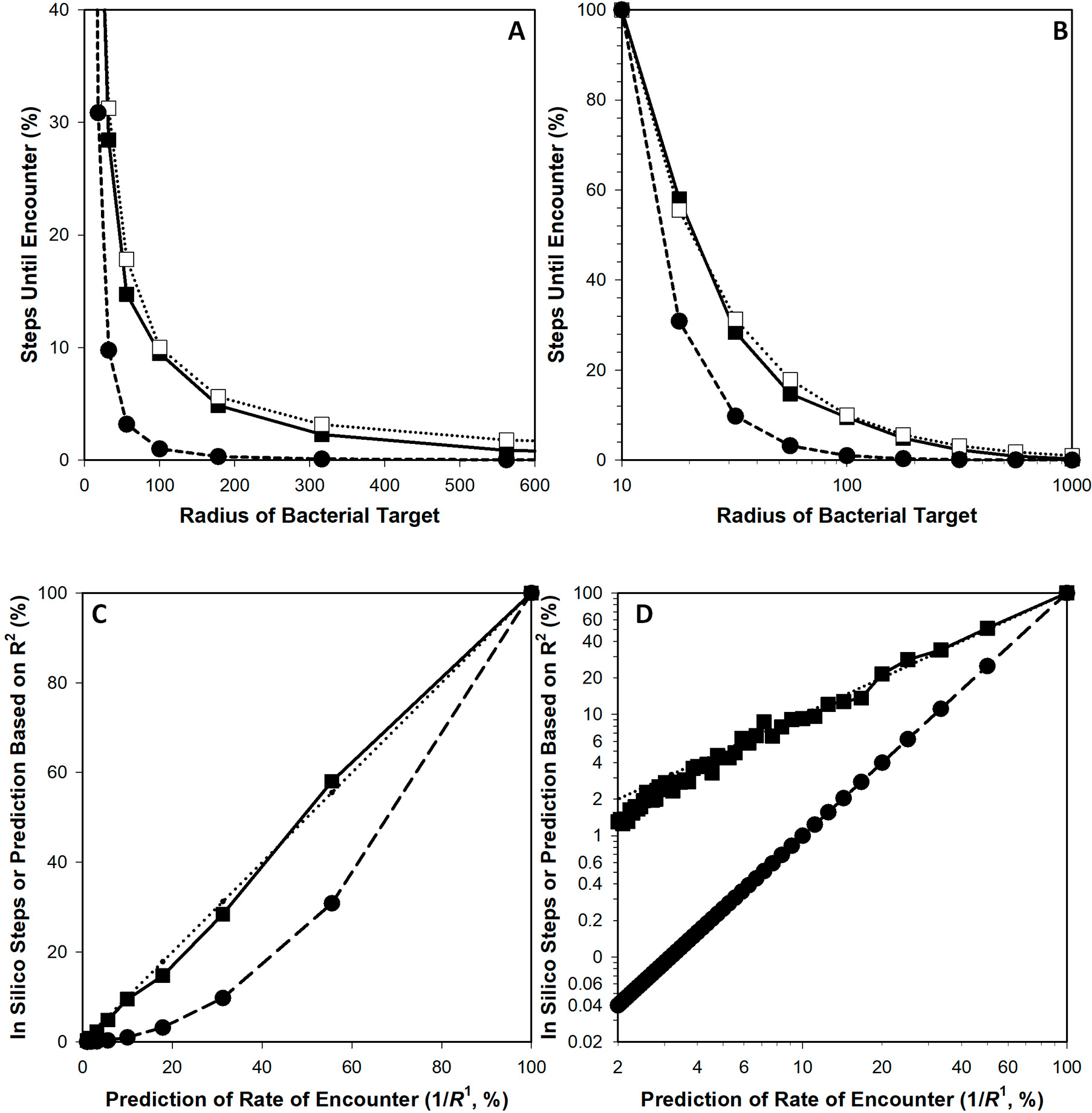
Comparison of $1/R^{2}$-based predictions with $1/R^{1}$-based predictions. These panels are otherwise identical to those presented in Figure 1, though with the scales of panels (**A**,**D**) modified. Upper panels (**A**,**B**): $1/R^{2}$-based predictions are shown using solid circles and dashed lines. $1/R^{1}$-based predictions are shown instead as open squares and dotted lines vs. solid squares and solid lines indicating simulation results. Lower panels (**C**,**D**): solid squares and solid lines are comparisons between simulation outcomes and $1/R^{1}$-based predictions, i.e., with the latter defining the x axis, whereas solid circles with dashed lines are comparisons between $1/R^{2}$-based predictions and $1/R^{1}$-based predictions. The $1/R^{1}$-derived dotted-line curves are again shown in these panels, also without symbols for the sake of clarity.

Comparisons were then made between using $1/R^{1}$ to predict relative number of steps until phage encounter with a bacterium vs. using $1/R^{2}$. The data and format of the resulting figure (Figure 3) are otherwise consistent with those presented in Figure 1. As can be seen, whereas $1/R^{1}$-based predictions (dotted lines in Figure 3A,B) are highly consistent with simulation outcomes (solid lines), $1/R^{2}$-based determinations (dashed lines) are somewhat less consistent with either.

## 4. Evidence for $R^{1}$vs. $R^{2}\;$from the Literature

Phage adsorption rate determinations have been undertaken now for at least nine decades [13,19]. Comparisons to theory have been less frequent, however, especially with varying of cell sizes. Here, I review one experiment which sought to test theory though providing at best only order-of-magnitude confirmation of Equation (1) (Section 4.1.1) and another which seems to compare adsorption rates with cell size (Section 4.1.2). In Section 4.2, I consider further theoretical support for $R^{1}$, as provided by Eriksen et al. [16]. I also use unit analysis, described also as dimensional analysis, to explore the validity of use of $R^{1}$ in Equation (1) (Section 4.3). There, though support is provided in terms of magnitudes, I point out that actual units of adsorption rate constants nonetheless are not necessarily equivalently described between studies.

### 4.1. Experimental Evidence?

#### 4.1.1. Comparing Adsorption Rates to Theory

Stent [10], p. 91 (references his) noted that, “Such calculations for optimal adsorption conditions of phage T4. . . show that the theoretical value of k/f and the experimental value of k are similar in magnitude; that is, f [as describing virion adsorption affinity for bacterial cells as used in Equation (1)] appears to be of the order of unity. In other words, nearly every collision between a T4 phage and an *E. coli* bacterium seems to result in fixation of the virus particle [19,25].” However, is that conclusion really true, or is it only an approximation? From the cited [25], f instead is calculated as 0.2, from which those authors (Stent and Wollman) concluded only that (p. 262), “An appreciable fraction of all random collisions between phage and host cell leads to irreversible union of the two.” It is possible that these differences between theory (derivations of k) and measures of actual adsorption rates could have been a consequence of k not being a function of $R^{1}$. Here, however, I suggest instead—as based on the presented in silico results along with further evidence provided below—that k likely was reasonably accurately derived by Stent and Wollman at least in terms of their representation of cell size. Therefore, under the experimental conditions employed [25], perhaps truly somewhat less than every actual phage encounter with a bacterium resulted in irreversible virion attachment.

A take-home message from the discussion found in the previous paragraph is that phage adsorption rates can vary not just with encounter rates, and therefore with cell size, along with virion diffusion rates (C), but also with phage affinity for a bacterium’s surface. Thus, to rigorously test how phage adsorption rates should vary with cell size, one must not only vary cell size but also keep phage affinity for those cells otherwise constant, presumably on a per-area basis of the cell’s surface. In particular, whatever serves as the phage receptor molecules, they should be present at the same density as well as availability across a bacterium’s surface regardless of that bacterium’s cell size. Such experimental testing is presented in the following section, though the potential for deviation from theory due to reduced affinities of phages for bacteria (f<1) persists there as well.

#### 4.1.2. Comparing Adsorption Rates to Cell Size

Hadas et al. [15] performed adsorption experiments employing phage T4 and a strain of its *Escherichia coli* host known as B/r, so named because it is more resistant to ultraviolet and X-ray radiation than its wild-type parent [26]. They grew the bacterium in media that was varied in terms of the carbon source present, e.g., acetate, glucose, glycerol, or succinate, but also casein hydrolysate, and the latter with glucose as well. In addition, glucose in combination with yeast extract was tested. They then determined various phage growth parameters such as adsorption rate, eclipse length, and latent period using standard methods [27,28]. Of particular relevance here, Hadas et al. present a graph of “Normalized adsorption rate” vs. “Total surface area” of “cells in the infected media”. The conclusion they reached from this exercise is that (p. 182), “This result indicates that the density over cell surface area of the receptor for the irreversible stage does not change with cell dimensions, which are modified by nutritional conditions.” See also [29] from the same group for prior analysis of that conclusion though not involving phages.

**Figure 4.**
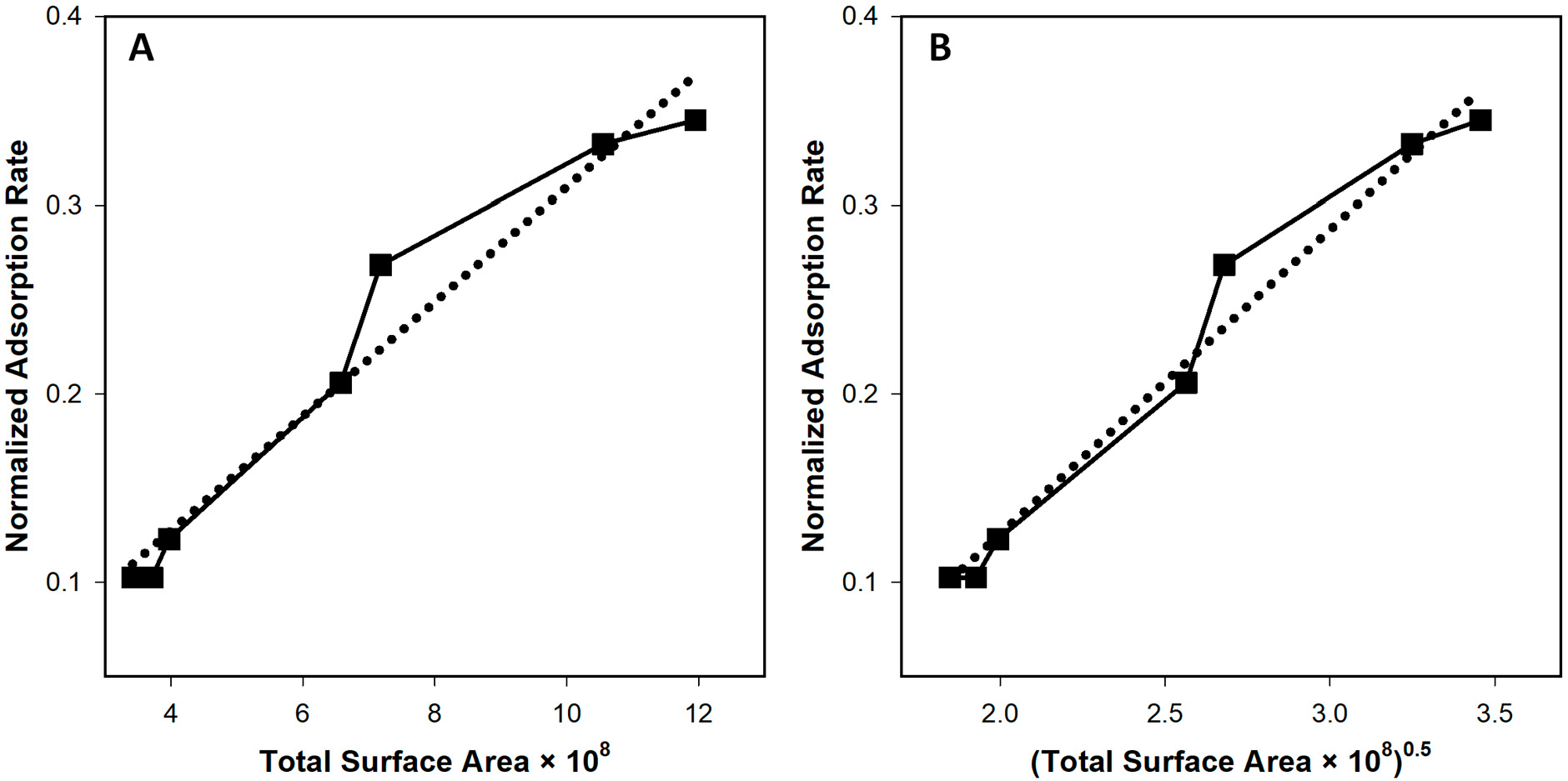
Reinterpretation of Figure 6 from Hadas et al. [15]. Panel (**A**) shows a representation of that figure as originally presented by Hadas et al. Panel (**B**) shows the same figure but with x axis shown as the square root of that presented in panel (**A**), thus $R^{2}$- and $R^{1}$-based going from panels (**A**) to (**B**). The least squares regressions (dotted lines) are as generated by SigmaPlot 15.0. As indicated in the main text, correlations coefficients are 0.9781 (for panel (**A**)) and 0.9878 (for panel (**B**)).

That same comparison as presented by Hadas et al. [15] leads to the possibility of $R^{1}$-contradicting results. Specifically, the figure described in the previous paragraph appears to indicate that a straight line can be drawn when comparing adsorption rates with cell surface area, which would seem to support phage adsorption rates varying with $R^{2}$. To better explore this possibility, I have extracted the data from their graph, using a snapshot from the publisher-supplied PDF which I then imported into Microsoft PowerPoint to manually determine point coordinates. There unquestionably is error in this analysis, though in terms of standard deviations of differences between axes units, this error likely is less than 3%. Furthermore, the slope, y intercept, and correlation coefficient as indicated in the original study were reported as 0.031, 0.004, and 0.98, respectively, whereas from the extracted data the numbers instead are 0.30, 0.006, and 0.98, respectively. See Figure 4A for this visualization of the extracted data as presented equivalently to that provided by Hadas et al.

Concerning Figure 1D from Section 3, the correlation coefficient is 0.9986. The calculation based on a comparison with $1/R^{2}$ rather than 1D’s $1/R^{1}$ yields instead a correlation coefficient of 0.9342 (no graph is shown for the latter). For Figure 1C, the correlation coefficients instead are 0.9990 (as relative to $1/R^{1}$, which is as shown) and 0.9604 (relative instead to $1/R^{2}$ and also not shown; see too the comparisons between correlation coefficients, r, shown in Table 1). Comparison by eye of the solid line (simulation results) and dashed line ($1/R^{2}$-based prediction) within Figure 3C,D furthermore is suggestive that these differences between $R^{1}$- and $R^{2}$-based curves are not trivial. This too appears to be the case in comparing Figure 4A,B, with their correlations coefficients the noted 0.9781 and 0.9878 for x equals total bacterial surface area, as proportional to $R^{2}$, vs. the square root of that surface, as proportional to $R^{1}$, respectively. Clearly, though, there is less of a difference between the $R^{1}$- and $R^{2}$-based curves generated from the Hadas et al. [15] data than with that provided by the model as presented in Section 3 (Figure 3C,D).

Alternatively, it is possible to re-examine this data using the square root of the provided area, i.e., thereby converting $R^{2}$ to $R^{1}$. This slightly improves the correlation coefficient, going from 0.9781 as calculated for Figure 4A when based on $R^{2}$ to instead 0.9878 as based on $R^{1}$ (see Figure 4B for the latter). Assuming that my interpretation of what the Hadas et al. [15] graph represents is correct, this would indicate that their results are at least as well explained based on an assumption that k varies with $R^{1}$ as with $R^{2}$, and objectively may be better explained by $R^{1}$. This analysis, though, does suggest that with relatively small differences in cell sizes (vs. the somewhat larger differences explored in Figures 1 and 2), both $R^{1}$ and $R^{2}$ seem to adequately describe variance in k with cell size, at least in terms of correlation coefficients. Whether that similarity would hold experimentally with larger differences in cell sizes such as multiple orders of magnitude, or in terms of differences in sizes of clusters of bacteria, both remain to be determined.

**Figure 5.**
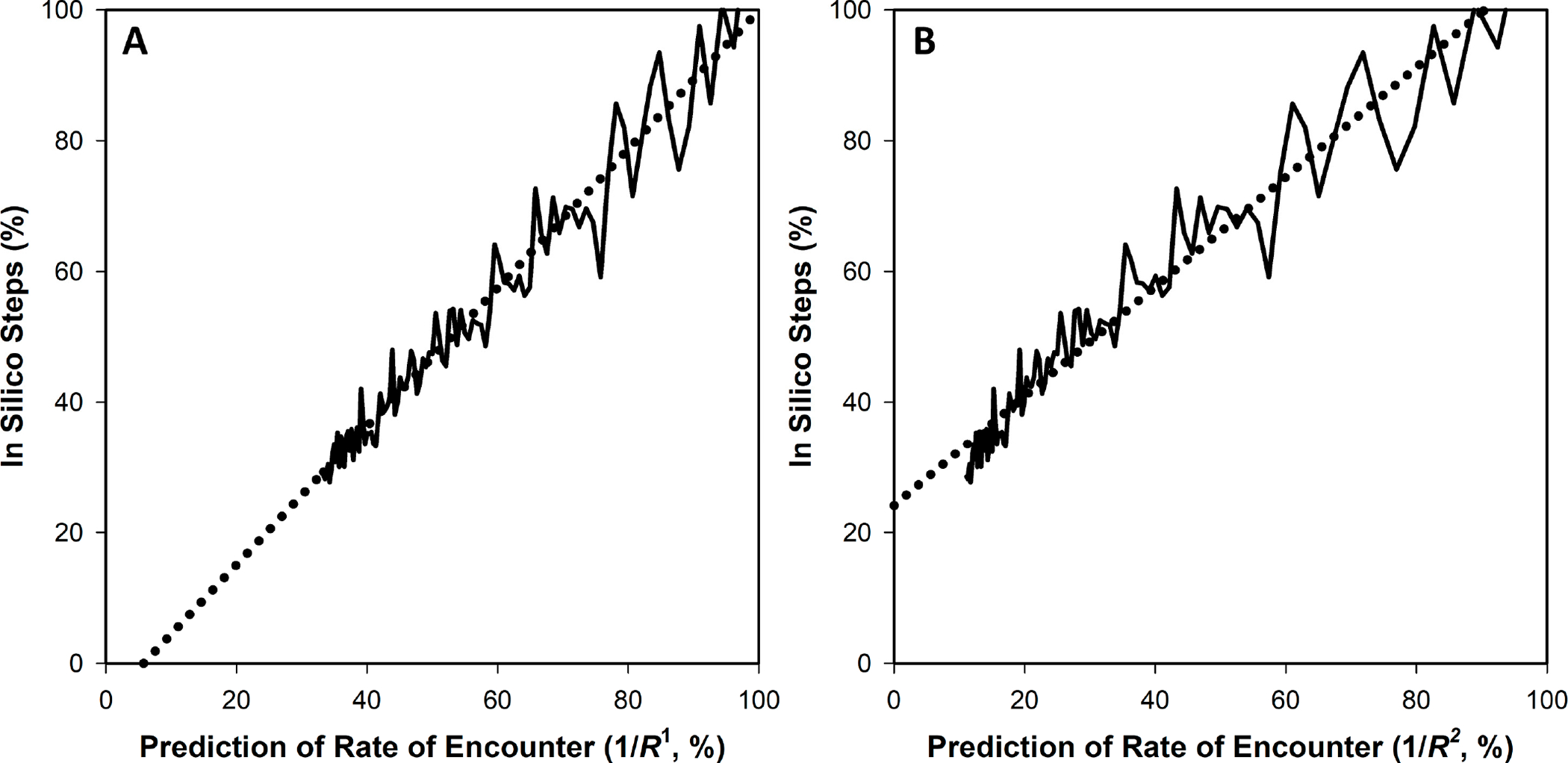
Simulations varying cell radius from 10 to 30 units. These two representations differ only in that panel (**A**) uses a prediction of $1/R^{1}$ whereas panel (**B**) uses a prediction of $1/R^{2}$ (thus $R^{1}$- and $R^{2}$-based going from panels (**A**) to (**B**)), with the data used to make the comparison considered in the main text as well as the first row of Table 2. As noted in the main text, these simulations consisted of cell sizes with radii incremented up in units of 0.2, for a total of 101 different sizes (for the sake of increased clarity the resulting symbols are not provided). Volumes in which virion diffusion occurs consisted of cubes of 500 units on a side, thus far exceeding the maximum cell radius of only 30 units, and 200 technical repeats were obtained per cell size, per simulation.

That differences between the effectiveness of $1/R^{1}$- and $1/R^{2}$-based predictions might be insubstantial given only small differences in cell sizes (previous paragraph) can be tested in silico using encounter.phage.org. For example, in incrementing cell sizes from 10 to 30 by units of 0.2, the resulting correlation coefficients are only, for example, 0.9763 (as a function of $1/R^{1}$) and 0.9706 (as a function of $1/R^{2}$), respectively, in a given test run (here, using 500 as the size of the volume, since cell sizes remain comparatively small and 200 technical repeats to reduce noise in the data; see Figure 5 for an example graph and Table 2 for a summary of multiple repeats of this simulation). This seems to suggest, along with the Hadas et al. data [15], that differentiating between the correlative power of $R^{1}$ vs. $R^{2}$ may be relevant particularly when exploring phage adsorption rates to clusters of bacteria which can be much larger than individual cells, an issue addressed the following section. The resulting slopes of the steps vs. predictions as shown in Figure 5 however are somewhat different, with that associated with $1/R^{1}$ (panel (**A**)) somewhat closer to 1.0 than that associated with $1/R^{2}$ (panel (**B**)). See the bottom rows of Table 2 for further exploration of that difference.

In this same study [15], treatment with penicillin had the effect of inhibiting cell division, resulting in cells that were “approximately four-fold larger”. Not surprisingly, this resulted in faster phage adsorption, though this did not result in four-fold faster adsorption. Rather, the ratio of adsorption rate constants with and without penicillin present appears to be only about 1.4 ≈ 4^0.25^. This contrasts with the theoretical expectations of Eriksen et al. [16] where instead 4^0.5^ (=2) to 4^0.8^ (≈3) is predicted. That difference between theory and experiment might indicate that penicillin had negatively affected phage affinity for the bacterial surface. It is of interest further that upon phage infection, burst sizes were found to be 2.5- to 4-fold larger with vs. without penicillin incubation, depending on the growth medium used [15]. This would appear to be particularly relevant in light of the concept known as phage-antibiotic synergy (PAS), in which improvements in phage growth parameters such as burst size can be seen in association with application of sub-inhibitory concentrations of antibiotic, as is often associated as well with increased cell length [12].

### 4.2. Adsorption to Rods and Spherical Clusters of Bacteria

Eriksen et al. [16] theoretically explored changes in adsorption rates as a function of both bacterial elongation and clustering into spherical microcolonies. This was performed in a manner similar to that provided here, though for spherical microcolonies the results are presented as a function of cell numbers rather than cluster radius (next paragraph). They noted for elongated bacteria, however, that “adsorption rate can be well approximated using only the surface area of the bacterial shape.” Their prediction of adsorption rate then employs an equation which takes the square root of this surface area value. That is, such that ${\left(R^{2}\right)}^{1/2}=R^{1}$. They then note that, “This approximated adsorption rate is remarkably close to the value we measure in our simulations.” (Quotations are from their Supplementary Materials). Thus, the theoretical evidence that adsorption rates to spherical bacteria scale to $R^{1}$ (Section 3) appears to be extendable to elongated bacteria as well.

In terms of spherical microcolonies, Eriksen et al. [16], also based on modeling, found a linear increase in rates of adsorption as a function of numbers of bacteria making up those microcolonies. This is shown using a log-log plot which yields a straight line (their Figure 5A). Focusing on the endpoint of their theoretical analysis, they find that a spherical microcolony consisting of 1000 (10^3^) bacteria is adsorbed at a rate that is only ten-fold greater than that of an isolated bacterium. A microcolony consisting of 10^3^ bacteria should have a volume that is 10^3^-fold greater than that of a single cell, assuming that the cells in microcolonies take up no more volume than when cells instead are isolated from other cells. Volume of course is a cubed function of radius, R, i.e., with the formula for the volume of a sphere equal to $4\pi R^{3}/3$. The cube-root of 10^3^ in turn is equal to 10, where the rate of adsorption to this same cluster as noted is predicted to be ten-times that of an individual bacterium. This then indicates that adsorption rates to these spherical bacterial clusters scale with $R^{1}$ (as the cube root of $R^{3}$) and this is rather than scaling with $R^{2}$, at least in their theoretical, also random walk-based analysis.

### 4.3. Unit Analysis

Phage adsorption rate constants (k) describe the likelihood that one phage will adsorb one bacterium as found within one unit volume of environment (such as 1 mL) over one unit of time (such as 1 min). Do these units (mL and min) support the prediction of $k\propto R^{1}$ vs., e.g., $k\propto R^{2}$?

Ignoring f, which is unitless, if we do a unit analysis for R×C=k, i.e., as from Equation (1), then we find the following [10]:

(4) $$\frac{cm}{1}\times \frac{{cm}^{2}}{min}=\frac{{cm}^{3}}{min}=\frac{mL}{min}.$$

The resulting k units are ${cm}^{3}/min$ [10,16,19], as equivalent to mL/min and based upon adsorption rate constants being defined in terms of milliliters and minutes. This makes more intuitive sense than k units of ${cm}^{4}/min$. That is, ${cm}^{4}/min$ would result from

(5) $$\frac{{cm}^{2}}{1}\times \frac{{cm}^{2}}{min}=\frac{{cm}^{4}}{min},$$

were we to use $R^{2}\left({cm}^{2}\right)$ instead of $R^{1}\left(cm\right)$ as the units describing bacterial size. Additionally, from Schlesinger [14], k can be defined as,

(6) $$k=\frac{2.3}{Nt}\log\frac{P_{0}}{P_{t}}=\frac{1}{Nt}\ln\frac{P_{0}}{P_{t}},$$

where N is bacterial concentration, $P_{0}$ is initial phage concentration, and $P_{t}$ is phage concentration at time, t, i.e., at a point after adsorption has begun to take place. The two P terms’ units cancel leaving 1/Nt. As N’s units are per volume, this results—using mL and min units—in k’s units again being proportional to unit volume per unit min.

Note, though, that even $k={cm}^{3}/min$ does not necessarily make intuitive sense. If we define k as the likelihood of adsorption given, as noted, the presence of one phage and one cell suspended in 1 mL (i.e., per mL) over one min of time (i.e., per min), then instead we might expect units of mL^−1^ min^−1^, which also can be units used for collision kernels more generally [22]: “number of collisions per unit volume and time”. That in fact is how k is sometimes expressed [15], including by this author [12]. Thus, while unit analysis provides us with at least an approximation of the expected units of k as based on $R^{1}$, are those units really the expected units of k, as the phage adsorption-rate constant? Or, instead, have k’s units—when presented as mL/min rather than as mL^−1^ min^−1^—been made to fit the above unit analysis rather than the actual units of adsorption likelihoods?

## 5. Discussion

The phage adsorption rate constant (k) collapses multiple aspects of what can be described as a phage virion’s extracellular search for bacteria, all into a single number. That approach is useful because, especially in the laboratory, it is fairly simple to determine k [9,27] and it also is possible to approximate the chemistry of in vivo adsorption environments in vitro, e.g., by employing body fluids such as serum or artificial urine [30,31]. It can be problematic to rely solely on such laboratory measurements, however, as bacteria often exist in forms for which determination of adsorption rate constants is less straightforward and experimental in situ determinations of phage adsorption rates are rarely undertaken. Of perhaps particular relevance, for the analyses presented and reviewed here, is the determination of rates that phages adsorb to bacteria that are clustered together rather than existing more in isolation [16], especially as it is common for clustered bacteria, as biofilms, to represent important components of bacterial infections [32–36].

The exercise presented here in Section 3 suggests that rates of phage encounter with bacteria, at least encounters with spherical bacteria, are proportional to $R^{1}$, i.e., simply R, and this is rather than, e.g., proportional to $R^{2}$. That is, the curves based on the simulations, presented as relative number of steps required for phages to reach bacteria, coincide nearly exactly with predicted curves based on an assumption of only $R^{1}$ defining cell size. Differences—as resulting in simulations vs. predictions not being quite identical (Figure 1)—presumably can be attributed especially to inherent simulation stochasticity (see Table 1 especially). Basing k on $R^{1}$ rather than on $R^{2}$ may be further supported by an exploration of unit analysis (Section 4.3). Experimental support also seems to exist for basing k on $R^{1}$, again rather than on $R^{2}$, though there the distinction is less substantial, perhaps because differences in cell sizes in those experiments were not large (Section 4.1.2). In addition is the theoretical work of Eriksen et al. [16], which again points to phage adsorption rates varying with $R^{1}$ rather than, e.g., with $R^{2}$. Lastly is the historical precedent for modeling the impact of bacterial size as a function of $R^{1}$ as provided by Schlesinger [13,14] in 1932 and see also Delbrück [19] from 1940.

A better understanding of how the size of bacterial clusters can impact their vulnerability to phage adsorptions may help to improve our ability to predict the potential for treatment phages to affect bacterial populations. This may be important particularly when targeted bacterial populations are difficult for applied phages to reach and thus difficult to dose directly with overwhelming phage numbers. At least with simpler shapes, our expectation therefore should be that cells or clusters which are two-fold larger in radius should also be two-fold more likely to be encountered by individual phages. Approximations of such clusters, dubbed as ‘aggregates’, can be associated for instance with chronic bacterial infections, such as *Pseudomonas aeruginosa* infecting the lungs of cystic fibrosis patients [17]. By default, we thus should assume that rates of phage encounter with those bacterial clusters should scale linearly as a function of their radii rather than with their surface areas, though with the caveat that adsorption rates may be slightly faster than this would predict for larger cells sizes.

Schlesinger’s theorizing, as based on the work of Smoluchowski [18], therefore seems to represent a successful application of mathematics to our understanding of phage biology. This, by extension, should be relevant to our ability to predict important aspects of the pharmacology of phage therapy, with a greater understanding of phage therapy pharmacology likely relevant to more widespread of adoption of this important antibacterial strategy [2].

## 6. Materials and Methods

Relevant details of the model used here are presented in the Section 3. For specific details, see instead Supplementary Materials Coding S1 for the JavaScript code used to run the presented simulation. See equivalently https://www.phage.org/encounter/steps.js (accessed on 14 August 2023).

## 7. Conclusions

Reviewed here is evidence that Schlesinger’s [13] 1932 theorizing that k is a function of $R\;(R^{1}$, rather than, e.g., $R^{2}$) likely is at least mostly correct. Consequently, we should expect that bacteria which are, e.g., twice as large in diameter, or spherical clusters of bacteria which similarly are twice as large in diameter, will also be twice as likely to be encountered by individual phage virions, rather than, e.g., four times as likely.

## Supplementary Material

JavaScript coding

## Figures and Tables

**Table 1.** Simulating phage encounters with bacterial sizes ranging from 10 to 1000 units in radius *.

	$\text{m}\;(x\;=\;1/R^{1})$	$\text{b}\;(x\;=\;1/R^{1})$	$r\;(x\;=\;1/R^{1})$	$\text{m}\;(x\;=\;1/R^{2})$	$\text{b}\;(x\;=\;1/R^{2})$	$r\;(x\;=\;1/R^{2})$

1	1.0184	−1.2667	0.9990	0.9861	8.4143	0.9604
2	0.9946	−1.1662	0.9992	0.9738	8.1196	0.9698
3	0.9973	−0.7747	0.9993	0.9729	8.5935	0.9664
4	0.9909	−3.3821	0.9933	0.9901	5.5440	0.9839
5	1.0311	−0.3799	0.9976	0.9880	9.5944	0.9477
6	0.9706	−1.6504	0.9948	0.9657	7.1596	0.9812
7	1.0590	0.8696	0.9896	0.9943	11.4469	0.9211
8	0.9735	−2.7446	0.9933	0.9735	6.0116	0.9848
9	1.0163	−0.9341	0.9992	0.9847	8.7232	0.9596
10	1.0296	−0.7564	0.9974	0.9909	9.1348	0.9515

Means:	1.0081	−1.2186	0.9963	0.9820	8.2742	0.9626
SDs:	0.0278	1.1905	0.0034	0.0097	1.7228	0.0196
*p*-values ^†^	0.0168	0.0000	0.0004	NA	NA	NA

* Abbreviations: m = slope, b = *y* intercept, *r* = correlation coefficient as based on a polynomial least-squares fit, SDs = standard deviations, NA = not applicable. Number of steps prior to encounter for each cell radius constitutes the y axis. Simulation parameter values used are equivalent to those employed in the simulations presented in Figure 1, panels (A-C), and Figure 2, panels (C,D). Actual simulations are graphed relative to $1/R^{2}$ in Figure 2, panels (C,D). The diffusion environment for these simulations was 2500 units on a side.

† Based on two-tailed Studenťs t-tests between $x\;=\;1/R^{1}$ and $x\;=\;1/R^{2}$ for m, b, and r, respectively.

**Table 2.** Simulating phage encounters with bacteria of sizes ranging from 10 to 30 units in radius *.

	$\text{m}\;(x\;=\;1/R^{1})$	$\text{b}\;(x\;=\;1/R^{1})$	$r\;(x\;=\;1/R^{1})$	$\text{m}\;(x\;=\;1/R^{2})$	$\text{b}\;(x\;=\;1/R^{2})$	$r\;(x\;=\;1/R^{2})$

1	1.0606	−6.1192	0.9763	0.8391	24.1075	0.9706
2	0.8794	−4.5956	0.9751	0.6966	20.4377	0.9707
3	1.0769	−4.4772	0.9790	0.8454	26.4352	0.9658
4	0.9266	−4.2082	0.9842	0.7338	22.1728	0.9796
5	0.9220	−5.9269	0.9785	0.7317	20.2775	0.9758
6	1.0330	−2.6378	0.9819	0.8133	26.9357	0.9715
7	1.0117	−3.7888	0.9852	0.7935	25.2762	0.9711
8	0.9541	−5.0865	0.9845	0.7559	22.0701	0.9802
9	1.0255	−7.0626	0.9852	0.8121	22.1242	0.9805
10	1.0546	−3.7976	0.9813	0.8272	26.4984	0.9673

Means:	0.9944	−4.7700	0.9811	0.7849	23.6335	0.9733
SDs:	0.0685	1.3081	0.0037	0.0517	2.5433	0.0054
*p*-values ^†^	0.0000	0.0000	0.0017	NA	NA	NA

* Abbreviations: m = slope, b = *y* intercept, *r* = correlation coefficient as based on a polynomial least-squares fit, SDs = standard deviations, NA = not applicable Number of steps prior to an encounter for each cell radius constitutes the y axis. Simulation parameter values used are equivalent to those employed in the simulations presented in Figure 5. Compare b, the y intercept, especially with the x-axis value of 100% seen in that figure.

† Based on two-tailed Student’s *t*-tests between $x\;=\;1/R^{1}$ and $x\;=\;1/R^{2}$ for m, b, and r, respectively.
