# Supplementary material for: Schlesinger Nailed It! Assessing a Key Primary Pharmacodynamic Property of Phages for Phage Therapy: Virion Encounter Rates with Motionless Bacterial Targets
Source: Drugs Drug Candidates. Author manuscript; Available in PMC 2024 May 20. (PMC11104014; doi:10.3390/ddc2030034)
Supplement: JavaScript coding [file NIHMS1981836-supplement-JavaScript_coding.zip › ddc-2509363-supplementary.pdf]

The following is the JavaScript coding used to support the presented simulations:

```
function createArray(length) {
    var arr = new Array(length || 0),
        i = length;

    if (arguments.length > 1) {
        var args = Array.prototype.slice.call(arguments, 1);
        while(i--) arr[length-1 - i] = createArray.apply(this, args);
    }

    return arr;
}

////////////////////////////////////////////////////////////////////////////////
// this function is responsible for movement within the space
// note that it works for all three or more dimensions, x, y, z
////////////////////////////////////////////////////////////////////////////////

function movement(current,maximum) {
    var newposition = -1;
    while (newposition < 0 || newposition > maximum) {
        // note that the following is ever so slightly biased toward 0; not sure how to get around that nor whether it matters given use of large spaces
        change = Math.round(Math.random());
        if (change == 0) {
            change = -1;
        }
        else {
            change = 1;
        }
    newposition = current + change;
    }
    return newposition;
}

////////////////////////////////////////////////////////////////////////////////
// this function takes the input values and delivers the output
////////////////////////////////////////////////////////////////////////////////
```

```
function Encounter() {

    var timestamp1 = Date.now();
    var today = new Date();
    var date = today.getFullYear()+'-'+(today.getMonth()+1)+'-'+today.getDate();
    var time = today.getHours() + ":" + today.getMinutes() + ":" + today.getSeconds();
    var dateTime1s = date+' '+time;
    var dateTime1p = date+'_'+time;

////////////////////////////////////////////////////////////////////////////////
// these just initialize the output; I'm artifically limiting to 41
////////////////////////////////////////////////////////////////////////////////



        result0.textContent = '';
        result1.textContent = '';
        result2.textContent = '';
        result3.textContent = '';
        result4.textContent = '';
        result5.textContent = '';
        result6.textContent = '';
        result7.textContent = '';
        result8.textContent = '';
        result9.textContent = '';
        result10.textContent = '';
        result11.textContent = '';
        result12.textContent = '';
        result13.textContent = '';
        result14.textContent = '';
        result15.textContent = '';
        result16.textContent = '';
        result17.textContent = '';
        result18.textContent = '';
        result19.textContent = '';
        result20.textContent = '';
        result21.textContent = '';
        result22.textContent = '';
```

```
        result23.textContent = '';
        result24.textContent = '';
        result25.textContent = '';
        result26.textContent = '';
        result27.textContent = '';
        result28.textContent = '';
        result29.textContent = '';
        result30.textContent = '';
        result31.textContent = '';
        result32.textContent = '';
        result33.textContent = '';
        result34.textContent = '';
        result35.textContent = '';
        result36.textContent = '';
        result37.textContent = '';
        result38.textContent = '';
        result39.textContent = '';
        result40.textContent = '';

/////////////////////////////////////////////////////////////////////////////////
// These are how the input information is obtained
/////////////////////////////////////////////////////////////////////////////////

    var sizespaceField = document.getElementById('sizespaceField').value;       // specifies the size of the adsorption environment
    var targetminField = document.getElementById('targetminField').value;       // specifies the initial size of the target, either radius or edge length
    var shapeField = document.getElementById('shapeField').value;               // specifies whether target is sphere (0) or cuboid (1)
    var cuboidField = document.getElementById('cuboidField').value;             // specifies if cuboid variable axis length
    var targetincsupField = document.getElementById('targetincsupField').value; // specifies total number of target sizes to explore
    var incrementField = document.getElementById('incrementField').value;       // specifies how target sizes will increment up, (+) for exponential, (-)
for linear increases
    var cornerField = 0;                                                        // specifies where the phage starts in environment with default being one corner (0)
                                                                                // did also have random insertion but for some reason no longer can get that work
    var dimensionsField = document.getElementById('dimensionsField').value;     // species whether 2D (2) or instead a 3D (3) search
    var repeatsField = document.getElementById('repeatsField').value;           // specifies number of technical repeats undertaken for each target size;
ultimate an average is provided as output
    var popupField = document.getElementById('popupField').value;               // specifies whether extended output (more than 10 target sizes) is
presented, particularly in a capturable form
```

```
////////////////////////////////////////////////////////////////////////////////
// begin various checks on that input data
////////////////////////////////////////////////////////////////////////////////

    if (sizespaceField.length < 1 || sizespaceField < 2) { // specifies how small the search environment can be; yes, a size of 2 is absurdly small
        sizespaceField = 2;
    }

    sizespaceField = Math.round(sizespaceField); // assures that variable is both a number and an integer

////////////////////////////////////////////////////////////////////////////////

    if (targetminField.length < 1 || targetminField > (sizespaceField/2)) { // makes sure that at least the minimum target size is at least half the size of the search environment, though this is assured again later in the program for all target sizes
        targetminField = Math.round(sizespaceField/2);
    }

    if (targetminField < 1) { // makes sure that the target size cannot be less than a discrete size of 1, which is basically the size of a phage
        targetminField = 1;
    }

    targetminField = Number(targetminField); // assures that the variable is a number

    targetminField = Math.round(targetminField); // assures that the variable is an integer

////////////////////////////////////////////////////////////////////////////////

    if (shapeField.length < 1 || shapeField < 0 || shapeField > 1) { // makes sure that only 0 and 1 are used to specify shapes
        shapeField = 0;
    }

    shapeField = Math.round(shapeField); // assures that variable is both a number and an integer

////////////////////////////////////////////////////////////////////////////////

    if (cuboidField.length < 1 || cuboidField < 0) { // makes sure that negative numbers not included with a return to the default value of 0 is so
        cuboidField = 0;
    }
```

```
    cuboidField = Math.round(cuboidField); // assures that variable is both a number and an integer

 ////////////////////////////////////////////////////////////////////////////////

    if (targetincsupField.length < 1 || targetincsupField < 1) { // setting default to 1 or greater
        targetincsupField = 1;
    }

    targetincsupField = Number(targetincsupField); // assures that the variable is a number

    targetincsupField = Math.round(targetincsupField); // assures that the variable is an integer

////////////////////////////////////////////////////////////////////////////////

    // If neg then linear incrementation. If pos, then exponential

var incrementExp = 1; // if linear then 0; if exponential then 1
var incvarsign = ""; // this default value specifies a positive value for presenting in popup windows

    if (incrementField.length < 1) {
        incrementField = 1; // defaulting value at 1
    }
    else {
        incrementField = Number(incrementField); // assures that the variable is a number


// I commented out the following so that I can do less-than-one increments
//
//        if (incrementField >= -1 && incrementField <= 1) {
//            incrementField = 1;   // defaulting value at 1
//            incrementExp = 0;
//        }

        if (incrementField < 0) { // I've changed this from -1 to 0 so that I can do less-than-one increments
            incrementField = Math.abs(incrementField);
            incrementExp = 0;
            incvarsign = "-";
```

```
        }
    }

////////////////////////////////////////////////////////////////////////////////

    if (cornerField.length < 1 || cornerField < 0 || cornerField > 1) { // makes sure that only 0 and 1 are used to specify shapes with 1 as default
        cornerField = 0;
    }

    cornerField = Math.round(cornerField); // assures that variable is both a number and an integer


////////////////////////////////////////////////////////////////////////////////

    if (dimensionsField.length < 1) { // keeps 2D as default if not specified
        dimensionsField = 2;
    }
    else {
        if (dimensionsField != 2 && dimensionsField != 3) { // keeps 2D as default if not properly specified
            dimensionsField = 2;
        }
    }

    dimensionsField = Math.round(dimensionsField); // assures that variable is both a number and an integer

////////////////////////////////////////////////////////////////////////////////

    if (repeatsField.length < 1 || repeatsField < 1) { // makes sure that at least one technical repeat is performed
        repeatsField = 1;
    }

    repeatsField = Math.round(repeatsField); // assures that variable is both a number and an integer

////////////////////////////////////////////////////////////////////////////////

    if (popupField.length < 1 || popupField < 0 || popupField > 1) { // makes sure that only 0 and 1 are used to specify shapes with 0 (no popup) as
default
        popupField = 0;
```

```
    }

    popupField = Math.round(popupField); // assures that variable is both a number and an integer

////////////////////////////////////////////////////////////////////////////////
// end various checks on that input data
////////////////////////////////////////////////////////////////////////////////

////////////////////////////////////////////////////////////////////////////////
// Just a bit of variable initializing
////////////////////////////////////////////////////////////////////////////////

var maxdimen = 0;

var Input = new Array(targetincsupField+1); // this is the target size, either radius or length of varying sized edge
var Output = new Array(targetincsupField+1); // this is the number of steps it takes for the phage to encounter the cell
var runs = 0;
var totalsteps = 0;
var squarevar = 0;
var numsteps = 0;
var centerx = 0;
var centery = 0;
var centerz = 0;

var startx = 0;
var starty = 0;
var startz = 0;
var centerx = 0;
var centery = 0;
var centerz = 0;
var endxmin = 0;
var endxmax = 0;
var endymin = 0;
var endymax = 0;
var endzmin = 0;
var endzmax = 0;
var xvar = 0;
var yvar = 0;
```

```
var zvar = 0;

var targetsize = 0;
var targetsizelocal = 0;
var targetvar = 0;
var targetvarlocal = 0;
var targetvaroutput = 0;

////////////////////////////////////////////////////////////////////////////////
// Defines placement of target, i.e., the bacterium
////////////////////////////////////////////////////////////////////////////////

var targetsize = 0; // a for loop incrementation variable

for (let targetsize = 0; targetsize < targetincsupField; targetsize++) { // initializing the arrays
   Input[targetsize] = 0;
   Output[targetsize] = 0;
   }

var Actualtotal = 0;
var InputtotalR1 = 0;
var InputtotalR2 = 0;
var InputmeanR1 = 0;
var InputmeanR2 = 0;
var Outputtotal = 0;
var Outputmean = 0;

targetsize = 0;

for (let targetsize = 0; targetsize < targetincsupField; targetsize++) {
   targetvar = 0;
   targetsizelocal = targetsizelocal + 1; // this apparently has to be here because targetsize will not work consistently in its stead, particularly below
for case 0

   switch(incrementExp) {
      case 0: targetvar = targetminField+(incrementField*(targetsizelocal-1)); break; // arithmetic increases
      case 1: targetvar = targetminField*Math.pow(incrementField,targetsizelocal-1); break; // exponential increases
   }
```

```
    if (targetvar > (sizespaceField/2)) { // this makes sure that the targets aren't too big
        targetvar = 0
    }
    else
    {
        targetvaroutput = targetvar;

//} // just making sure I know where this otherwise would be

// 11111111111111111111111111111111 (start) // just helping to keep track of what's inside of what

runs = 0; // a for loop incrementation variable
totalsteps = 0; // this keeps track of the sum of steps across multiple technical repeats

for (let runs = 0; runs < repeatsField; runs++) { // this increments up target-cell sizes

// 22222222222222222222222222222222 (start) // just helping to keep track of what's inside of what

////////////////////////////////////////////////////////////////////////////////
// Defines placement of target, i.e., the bacterial cell
////////////////////////////////////////////////////////////////////////////////

startx = 0; starty = 0; startz = 0;         // these are where the phages start their diffusion
centerx = 0; centery = 0; centerz = 0;      // these are the centers of the diffusion environments; formerly for some reason I had called thse just end, perhaps to go with the following
endxmin = 0; endxmax = 0;                   // these are smaller and larger numbers defing squares or rectangles (x and y) or cubes or rectangular cuboids (x, y, and z)
endymin = 0; endymax = 0;
endzmin = 0; endzmax = 0;

centerx = Math.round(sizespaceField/2)-1;
centery = Math.round(sizespaceField/2)-1;
centerz = Math.round(sizespaceField/2)-1

squarevar = targetvar; // this is here to make sure that a squares or cubes have an edge length of only targetvar so do not become rectangular cuboids
```

```
if (shapeField == 1 && cuboidField != 0) { // this is here to make sure that a rectangular cuboid varies during runs only on the x axis, i.e., only with
targetvar
    squarevar = cuboidField;
}

if (targetvar % 2 == 0 && targetvar > 1) {
//    result1.textContent = 'even';
    endxmin = centerx - Math.round(targetvar/2) + 1;
    endxmax = centerx + Math.round(targetvar/2) + 0;
    endymin = centery - Math.round(squarevar/2) + 1;
    endymax = centery + Math.round(squarevar/2) + 0;
    endzmin = centerz - Math.round(squarevar/2) + 1;
    endzmax = centerz + Math.round(squarevar/2) + 0;
}
else {
//    result1.textContent = 'odd';
    endxmin = centerx - Math.round((targetvar-1)/2);
    endxmax = centerx + Math.round((targetvar-1)/2);
    endymin = centery - Math.round((squarevar-1)/2);
    endymax = centery + Math.round((squarevar-1)/2);
    endzmin = centerz - Math.round((squarevar-1)/2);
    endzmax = centerz + Math.round((squarevar-1)/2);
  }

if (targetvar == 1 && cuboidField <= 1) { // this should just make sure that min size is equal to 1 unit in all dimensions
    endxmin = centerx;
    endxmax = centerx;
    endymin = centery;
    endymax = centery;
    endzmin = centerz;
    endzmax = centerz;
}

if (targetvar == 1 && cuboidField > 1) {
    endxmin = centerx;
    endxmax = centerx;
    if (squarevar % 2 == 0 && squarevar > 1) {
        endymin = centery - Math.round(squarevar/2) + 1;
```

```
        endymax = centery + Math.round(squarevar/2) + 0;
        endzmin = centerz - Math.round(squarevar/2) + 1;
        endzmax = centerz + Math.round(squarevar/2) + 0;
        }
    else {
        endymin = centery - Math.round((squarevar-1)/2);
        endymax = centery + Math.round((squarevar-1)/2);
        endzmin = centerz - Math.round((squarevar-1)/2);
        endzmax = centerz + Math.round((squarevar-1)/2);
    }
}

if (dimensionsField == 2) {
    startz = 0;
    centerz = 0;
    endzmin = 0;
    endzmax = 0;
}

////////////////////////////////////////////////////////////////////////////////
// Determines where to start the simulation, default is in corner
////////////////////////////////////////////////////////////////////////////////

maxdimen = sizespaceField - 1; // this is set to -1 because sizespaceField is specified as a 1 to # rather than 0 to #, e.g., 250 rather than 249

if (cornerField == 0) { // this is default, in corner
    startx = 0;
    starty = 0;
    startz = 0;
}

xvar = startx;
yvar = starty;
zvar = startz;

////////////////////////////////////////////////////////////////////////////////
// Runs single round of random walk
////////////////////////////////////////////////////////////////////////////////
```

```
numsteps = 0;
targetvarlocal = Number(targetvar); // this is here just for the sake of debugging

if (dimensionsField == 2) {
    centerz = 0;
}

if (targetvar == 1 && cuboidField <= 1) { // this is used only if the target is a single unit in all dimensions
    while (xvar != centerx || yvar != centery || zvar != centerz) {
        whichone = Math.round((dimensionsField-1)*Math.random());
        switch(whichone) {
            case 0: xvar = movement(xvar,maxdimen); break;
            case 1: yvar = movement(yvar,maxdimen); break;
            case 2: zvar = movement(zvar,maxdimen); break;
        }
        numsteps = numsteps + 1;
    }
}
else {
    switch(shapeField) {
        case 0: /////////// if target is a circle or sphere /////////////////
            do {
                whichone = Math.round((dimensionsField-1)*Math.random());
                switch(whichone) {
                    case 0: xvar = movement(xvar,maxdimen); break;
                    case 1: yvar = movement(yvar,maxdimen); break;
                    case 2: zvar = movement(zvar,maxdimen); break;
                }
                numsteps = numsteps + 1;
            }   while (Math.pow(Math.pow(xvar-centerx,2)+Math.pow(yvar-centery,2)+Math.pow(zvar-centerz,2),0.5)>targetvarlocal);
            break;
        case 1: /////////// if target is a square or cube ///////////////////
            do {
                whichone = Math.round((dimensionsField-1)*Math.random());
                switch(whichone) {
                    case 0: xvar = movement(xvar,maxdimen); break;
                    case 1: yvar = movement(yvar,maxdimen); break;
```

```
                    case 2: zvar = movement(zvar,maxdimen); break;
                }
                numsteps = numsteps + 1;
            } while (!(xvar >= endxmin &&  xvar <= endxmax && yvar >= endymin && yvar <= endymax && zvar >= endzmin && zvar <= endzmax));
    break;
    }
}

////////////////////////////////////////////////////////////////////////////////
////////////////////////////////////////////////////////////////////////////////

totalsteps = totalsteps + numsteps;

// 22222222222222222222222222222222 (end)

}

// 11111111111111111111111111111111 (start)

}

////////////////////////////////////////////////////////////////////////////////
// Saves mean number of steps to reach target
////////////////////////////////////////////////////////////////////////////////

Input[targetsizelocal] = targetvar;
Output[targetsizelocal] = totalsteps/repeatsField;


////////////////////////////////////////////////////////////////////////////////
// Following are needed to generate subsequent statistics
////////////////////////////////////////////////////////////////////////////////

if (targetvar > 0) {
    InputtotalR1 = InputtotalR1 + (1/Math.pow(Input[targetsizelocal],1));
    InputtotalR2 = InputtotalR2 + (1/Math.pow(Input[targetsizelocal],2));
    Outputtotal = Outputtotal + Output[targetsizelocal];
    Actualtotal = Actualtotal + 1;
```

```
    }

}

////////////////////////////////////////////////////////////////////////////////
// Below is for generation of slope, y-int, and correlation coeficient for R^1
////////////////////////////////////////////////////////////////////////////////

var sumproddif = 0;
var sumsqinput = 0;
var sumsqoutput = 0;
var SumIO = 0;
var SumI = 0;
var SumO = 0;
var SumI2 = 0;
var InputActual = 0;
var OutputActual = 0;
var Actual = 0;

if (Actualtotal > 0) {
    InputmeanR1 = 100*(InputtotalR1/Actualtotal)/(1/Math.pow(Input[1],1));
    InputmeanR2 = 100*(InputtotalR2/Actualtotal)/(1/Math.pow(Input[1],2));
    Outputmean = 100*(Outputtotal/Actualtotal)/Output[1];

    for (let targetsize = 0; targetsize < targetincsupField; targetsize++) {
        Actual = Actual + 1;
        InputActual = 100*(1/Math.pow(Input[Actual],1))/(1/Math.pow(Input[1],1));
        OutputActual = 100*Output[Actual]/Output[1];

        if (Actual <= Actualtotal) {
            SumIO = SumIO + (InputActual*OutputActual);
            SumI = SumI + InputActual;
            SumO = SumO + OutputActual;
            SumI2 = SumI2 + Math.pow(InputActual,2);
            sumproddif = sumproddif + ((InputActual - InputmeanR1)*(OutputActual - Outputmean));
            sumsqinput = sumsqinput + Math.pow(InputActual - InputmeanR1,2);
            sumsqoutput = sumsqoutput + Math.pow(OutputActual - Outputmean,2);
        }
```

```
    }

var R1Slope = ((Actualtotal*SumIO) - (SumI*SumO))/((Actualtotal*SumI2)-Math.pow(SumI,2));
// var R1Slope = sumproddif/sumsqinput; // this is an alt way to do the above
var R1Intercept = (SumO - (R1Slope*SumI))/Actualtotal
var R1Correl = sumproddif/Math.pow(sumsqinput*sumsqoutput,0.5);

////////////////////////////////////////////////////////////////////////////////
// Below is for generation of slope, y-int, and correlation coeficient for R^2
////////////////////////////////////////////////////////////////////////////////

sumproddif = 0;
sumsqinput = 0;
sumsqoutput = 0;
SumIO = 0;
SumI = 0;
SumO = 0;
SumI2 = 0;
InputActual = 0;
OutputActual = 0;
Actual = 0;

    for (let targetsize = 0; targetsize < targetincsupField; targetsize++) {
        Actual = Actual + 1;
        InputActual = 100*(1/Math.pow(Input[Actual],2))/(1/Math.pow(Input[1],2));
        OutputActual = 100*Output[Actual]/Output[1];

        if (Actual <= Actualtotal) {
            SumIO = SumIO + (InputActual*OutputActual);
            SumI = SumI + InputActual;
            SumO = SumO + OutputActual;
            SumI2 = SumI2 + Math.pow(InputActual,2);
            sumproddif = sumproddif + ((InputActual - InputmeanR2)*(OutputActual - Outputmean));
            sumsqinput = sumsqinput + Math.pow(InputActual - InputmeanR2,2);
            sumsqoutput = sumsqoutput + Math.pow(OutputActual - Outputmean,2);
        }
```

```
    }

var R2Slope = ((Actualtotal*SumIO) - (SumI*SumO))/((Actualtotal*SumI2)-Math.pow(SumI,2));
// var R2Slope = sumproddif/sumsqinput; // this is an alt way to do the above
var R2Correl = sumproddif/Math.pow(sumsqinput*sumsqoutput,0.5);
var R2Intercept = (SumO - (R2Slope*SumI))/Actualtotal

    }

//////////////////////////////////////////////////////////////////////////////
// Below outputs results to screen
//////////////////////////////////////////////////////////////////////////////

    result1.textContent = '------------------------------------------------------------------------------';

     if (dimensionsField == 2) {
        result2.textContent = 'Square environ edge length = ' + (maxdimen + 1) + ' (0 to ' + (maxdimen) +' units)';
    }
    if (dimensionsField == 3)  {
        result2.textContent = 'Cube environ edge length = ' + (maxdimen + 1) + ' (0 to ' + (maxdimen) +' units)';
    }

    result3.textContent = 'Target dimensions explored: min = ' + targetminField + ', max = ' + Math.round(100*targetvaroutput)/100;

    result4.textContent = 'Following are results from last round run only:';

    switch(shapeField) {
        case 0: {
            result5.textContent = '-- starting x = ' + startx + ', center x = ' + centerx + ', ending x = ' + xvar;
            result6.textContent = '-- starting y = ' + starty + ', center y = ' + centery + ', ending y = ' + yvar;

            if (dimensionsField == 3) {
                result7.textContent = '-- starting z = ' + startz + ', center z = ' + centerz + ', ending z = ' + zvar;
            }

            result8.textContent = '-- final phage distance from center = ' + Math.round(100*Math.pow(Math.pow(xvar-centerx,2)+Math.pow(yvar-
centery,2)+Math.pow(zvar-centerz,2),0.5))/100;
```

```
            if (dimensionsField == 2) {
                result9.textContent = '-- [ calc: (' + Math.round(100*Math.pow(xvar-centerx,2))/100 + ' + ' + Math.round(100*Math.pow(yvar-
centery,2))/100 + ')^0.5 = ' + Math.round(100*Math.pow(Math.pow(xvar-centerx,2)+Math.pow(yvar-centery,2)+Math.pow(zvar-centerz,2),0.5))/100 +
' ]';
            }

            if (dimensionsField == 3) {
                result9.textContent = '-- [ calc: (' + Math.round(100*Math.pow(xvar-centerx,2))/100 + ' + ' + Math.round(100*Math.pow(yvar-
centery,2))/100 + ' + ' + Math.round(100*Math.pow(zvar-centerz,2))/100 + ')^0.5 = ' + Math.round(100*Math.pow(Math.pow(xvar-
centerx,2)+Math.pow(yvar-centery,2)+Math.pow(zvar-centerz,2),0.5))/100 + ' ]';
            }

            result10.textContent = '-- thus, at end of last run, ' + Math.round(100*Math.pow(Math.pow(xvar-centerx,2)+Math.pow(yvar-
centery,2)+Math.pow(zvar-centerz,2),0.5))/100 + ' <= ' + Math.round(100*targetvaroutput)/100 + ' = R';
        } break;
        case 1: {
            result5.textContent = '-- start x = ' + startx + ', center x = ' + centerx + ', final x position = ' + xvar;
            result6.textContent = '-- start y = ' + starty + ', center y = ' + centery + ', final y position = ' + yvar;
            if (dimensionsField == 3) {
                        result7.textContent = '-- startz = ' + startz + ', center z = ' + centerz + ', final z position = ' + zvar;
            }
            result8.textContent = '-- target x location (max units): min = ' + endxmin + ', max = ' + endxmax;
            result9.textContent = '-- target y location (max units): min = ' + endymin + ', max = ' + endymax;
            result10.textContent = '-- target z location (max units): min = ' + endzmin + ', max = ' + endzmax;
        } break;
    }

    result11.textContent = '-- ave # steps \'til encounter = ' + Math.round(100*totalsteps/repeatsField)/100 + ' (' +
Number.parseFloat(totalsteps/repeatsField).toExponential(2) + ')';

    result12.textContent = '------------------------------------------------------------------';

    result13.textContent = 'Steps f(1/R^1): m = ' + String((Math.round(10000*R1Slope)/10000)) + ', b = ' +
String((Math.round(10000*R1Intercept)/10000)) + ', r = ' + String((Math.round(10000*R1Correl)/10000));
    result14.textContent = 'Steps f(1/R^2): m = ' + String((Math.round(10000*R2Slope)/10000)) + ', b = ' +
String((Math.round(10000*R2Intercept)/10000)) + ', r = ' + String((Math.round(10000*R2Correl)/10000));

    if (incrementField != 0) {
```

```
        result15.textContent = 'First ten outputs are shown as follows:';
        result16.textContent = '(set Popup = 1 & Increm to > 10 for greater range)';
    }

    targetminField = Number(targetminField);
    incrementField = Number(incrementField);

    function ScrOutputStr (Outputvar) {
        Outputvar = Number(Outputvar+1);
        if (Input[Outputvar] > 0) {
            OutputString = 'Targ. size: ' + String(Math.round(10*Input[Outputvar])/10) + ', steps: ' + String(Math.round(10*Output[Outputvar])/10) + ' (' +
String(Number.parseFloat(Output[Outputvar]).toExponential(2)) + ') = ' + String(Math.round(100*100*(Output[Outputvar])/(Output[1]))/100) + '%';
        }
        else {
            if (Outputvar <= targetincsupField) {
                OutputString = 'NOTE: target dimension was > 1/2 space dimension';
            }
            else {
                OutputString = ' ';
            }
        }
        return OutputString;
    }

    result17.textContent = ScrOutputStr(0);
    result18.textContent = ScrOutputStr(1);
    result19.textContent = ScrOutputStr(2);
    result20.textContent = ScrOutputStr(3);
    result21.textContent = ScrOutputStr(4);
    result22.textContent = ScrOutputStr(5);
    result23.textContent = ScrOutputStr(6);
    result24.textContent = ScrOutputStr(7);
    result25.textContent = ScrOutputStr(8);
    result26.textContent = ScrOutputStr(9);

    if (incrementField != 0) {
        result25.textContent = '----------------------------------------------------------------------------';
```

```
    var timestamp2 = Date.now();
    date = today.getFullYear()+'-'+(today.getMonth()+1)+'-'+today.getDate();
    time = today.getHours() + ":" + today.getMinutes() + ":" + today.getSeconds();
    dateTime2s = date+' '+time;
    dateTime2p = date+'_'+time;

    result27.textContent = "Date and time of run completion: " + dateTime1s;
    result28.textContent = "Date and time of run completion: " + dateTime2s;
    result29.textContent = "Run length in seconds: " + Math.round(1000*(timestamp2-timestamp1))/1000/1000;
    result30.textContent = "Run length in minutes: " + Math.round(1000*(timestamp2-timestamp1)/60)/1000/1000;

    }

////////////////////////////////////////////////////////////////////////////////
// Below outputs results to popup window
////////////////////////////////////////////////////////////////////////////////

if (popupField == "1") {
    var myWindow = window.open("", "MsgWindow", "width=700,height=600");

    myWindow.document.write("<p>");
    myWindow.document.write(String(dateTime2p) + "_(date_and_time_of_run_completion)<BR>");
    myWindow.document.write(String(sizespaceField) + "_=_sizespace_(1_dimension_of_environment_size)<BR>");
    myWindow.document.write(String(targetminField) + "_=_targetmin_(starting_dimension_of_target_size)<BR>");
    myWindow.document.write(String(shapeField) + "_=_shape_(0_is_circle_or_sphere,_1_is_square_or_cube)<BR>");
    myWindow.document.write(String(targetincsupField) + "_=_targetincsup_(number_of_lines_of_output)<BR>");
    myWindow.document.write(incvarsign + String(incrementField) + "_=_increment_(1_is_+1_incr;_>1_is_exp_incr;_<-1_is_arith_incr)<BR>");
    myWindow.document.write(String(cornerField) + "_=_corner_(1_=_start_in_corner;_0_=_random_start_point)<BR>");
    myWindow.document.write(String(dimensionsField) + "_=_dimensions_(2D_or_3D_random_walks)<BR>");
    myWindow.document.write(String(repeatsField) + "_=_repeats_(number_of_technical_repeats)<BR>");
    myWindow.document.write(String(dateTime1p) + "_=_date_and_time_of_start<BR>");
    myWindow.document.write(String(dateTime2p) + "_=_date_and_time_of_completion<BR>");
    myWindow.document.write(String((timestamp2-timestamp1)/1000) + "_=_seconds_run_time<BR>");
    myWindow.document.write(String((timestamp2-timestamp1)/1000/60) + "_=_minutes_run_time<BR>");
    myWindow.document.write(String((timestamp2-timestamp1)/1000/60/60) + "_=_hours_run_time<BR>");
    myWindow.document.write("<BR>");
```

```
    myWindow.document.write("Steps_function_1/R^1:_m_=_" + String((Math.round(10000*R1Slope)/10000)) + ",_b_=_" +
String((Math.round(10000*R1Intercept)/10000)) +  ",_r_=_" + String((Math.round(10000*R1Correl)/10000)));
    myWindow.document.write("<BR>");
    myWindow.document.write("Steps_function_1/R^2:_m_=_" + String((Math.round(10000*R2Slope)/10000)) + ",_b_=_" +
String((Math.round(10000*R2Intercept)/10000)) +  ",_r_=_" + String((Math.round(10000*R2Correl)/10000)));

    myWindow.document.write("<BR><BR>");
    myWindow.document.write("targetsize numbersteps %steps<BR>");
    myWindow.document.write("<BR>");

    targetsizelocal = 0;

    var targetsize = 0;

    for (let targetsize = 0; targetsize < targetincsupField; targetsize++) {
        targetsizelocal = targetsizelocal + 1
        if (Input[targetsizelocal] > 0) {
            myWindow.document.write(Input[targetsizelocal] + " " + Math.round(10000*Output[targetsizelocal])/10000 + " | " +
Math.round(100*10000*((1/Input[targetsizelocal])/(1/Input[1])))/10000 + " " + Math.round(100*10000*(Output[targetsizelocal])/(Output[1]))/10000 + "
% " + Math.round(100*10000*((1/Math.pow(Input[targetsizelocal],2))/(1/Math.pow(Input[1],2))))/10000 + " " +
Math.round(100*10000*(Output[targetsizelocal])/(Output[1]))/10000 + " " + "%<BR>");
        }
        else {
            myWindow.document.write("target_dimension_&gt;_1/2_space_dimension<BR>");
        }
    }
    myWindow.document.write("</p>");
}

}

// use an eventlistener for the event
var subButton1 = document.getElementById('subButton1');
subButton1.addEventListener('click', Encounter, false);
```
